# The profile of key gut microbiota members and short-chain fatty acids in patients with sepsis

**DOI:** 10.1016/j.heliyon.2023.e17880

**Published:** 2023-07-01

**Authors:** Edris Nabizadeh, Javid Sadeghi, Mohammad Ahangarzadeh Rezaee, Hamed Hamishehkar, Alka Hasani, Hossein Samadi Kafil, Yaghoob Sharifi, Solmaz Asnaashari, Hiva Kadkhoda, Reza Ghotaslou

**Affiliations:** a Infectious and Tropical Diseases Research Center, Tabriz University of Medical Sciences, Tabriz, Iran; b Department of Bacteriology and Virology, Faculty of Medicine, Tabriz University of Medical Sciences, Tabriz, Iran; c Infectious and Tropical Diseases Research Center, Tabriz University of Medical Sciences and Department of Laboratory Sciences, Faculty of Paramedicine, Tabriz University of Medical Sciences, Tabriz, Iran; d Drug Applied Research Center, Tabriz University of Medical Sciences, Tabriz, Iran; e Department of Microbiology, Faculty of Medicine, Urmia University of Medical Sciences, Urmia, West Azerbaijan, Iran; f Biotechnology Research Center, Tabriz University of Medical Sciences, Tabriz, Iran

**Keywords:** Gut microbiota, Sepsis, Short-chain fatty acids, Faecalibacterium prausnitzii, Bifidobacterium sp

## Abstract

Sepsis is a complex clinical disorder with heterogeneous etiological factors. Given its high mortality rate, it is considered a global health issue. Recently, the link between gut microbiota and their metabolites, especially short-chain fatty acids, in the pathophysiology of sepsis has been reported. However, there are few findings to confirm this relationship. This study aimed to evaluate some key gut microbiota members, pathogenic bacteria, and short-chain fatty acids in non-ICU patients with sepsis caused by bacteremia compared to a control group. In this case-control study, 45 stool samples from patients with sepsis and 15 healthy persons were collected from October 2021 to August 2022 in Tabriz, Iran. The position of some gut microbiota members and the main short-chain fatty acids concentration were assessed in the two groups by the Q-PCR and the high-performance liquid chromatography system. *Faecalibacterium prausnitzii* and Bifidobacterium sp. As bacterial with protective features in non-ICU patients with sepsis decreased significantly. Moreover, the concentrations of acetic acid and propionic acid significantly decreased in this group compared to the healthy volunteers. In contrast, the pathogenic bacteria members such as Enterobacteriaceae and *Bacteroides* sp. Increased significantly in the patients compared to the healthy individuals. The concentration of butyric acid decreased in the patients, but this change was not significant in the two groups. Protective and immune functions of *F. prausnitzii* and *Bifidobacterium* sp., as well as acetate and propionate, are evident. In this investigation, this profile was significantly reduced in non-ICU patients with sepsis compared to the control group.

## Highlights

• Sepsis is a complex clinical disorder with heterogeneous etiological factors. Recently, the link between gut microbiota and their metabolites, especially short-chain fatty acids, in the pathophysiology of sepsis has been reported. However, there are few findings to confirm this relationship.
• In this study, some key members of the gut microbiota as well as some pathogenic bacteria were different in the healthy and patient groups.
• Acetic and propionic acid, as the most important metabolites of intestinal microbiota function, decreased dramatically in the group of patients.
• The modified microbiome and its metabolites may be helpful for the management of patients with sepsis. The efficacy of this strategy will be proven by conducting more studies to understand the GM profile in these patients.

## 1 Introduction

Sepsis, as a complex clinical disorder with heterogeneous etiological factors, is caused by an imbalanced response of the host to infectious agents, resulting in multiple organ dysfunction and high mortality rates [1]. According to the available evidence, 31.5 million cases of sepsis occur each year in the world, of which 19.4 million cases have severe sepsis, and the total death rate is about 5.3 million [2]. Therefore, considering this mortality rate, sepsis is still considered a global health issue. Irregular responses to pro-inflammatory situations caused by infectious agents are considered the clear pathophysiology aspect of sepsis. Moreover, recent studies have shown that sepsis patients have a disorder in the body's homeostasis and suffer from suppressed cellular immunity in this condition [3,4]. On the other hand, in recent years, the link between gut microbiota (GM) and their metabolites, especially short-chain fatty acids (SCFAs), in the pathophysiology of sepsis has been highlighted [5].

The GM has the largest and most heterogeneous microbial community, constituting a complex ecosystem with trillions of bacteria as the dominant population among other microorganisms. Healthy GM plays a critical role in host health, and its function is generally discussed in the literature from three points of view, including metabolic, immune, and protective aspects [6]. An imbalance in this complex microbial ecosystem, called dysbiosis, plays a role in the etiology of sepsis [7]. A case-control study on 20 sepsis patients and 20 healthy cases confirmed severe dysbiosis [8]. On the other hand, acetic, propionic, and butyric acids are the main SCFAs resulting from the metabolic fermentation of gut microbiota members [9]. Overall, SCFAs have various functions in the human intestine, such as providing basic energy for the proliferation of intestinal epithelial cells, maintaining the homeostasis of the intestinal mucosa, playing a role in the local and systemic immune system of the intestine, exerting anti-inflammatory properties and controlling inflammation [10], plus affecting pathogenic bacteria by releasing reactive oxygen species. Therefore, the concentration of disturbed SCFAs can be considered another etiological factor for sepsis. In critically ill patients with sepsis, the concentration of acetic, propionic, and butyric acid was significantly disturbed compared to the healthy group [11].

Most studies addressed GM composition in critically ill patients with sepsis in the ICU, and there is also limited information on SCFAs profiles in patients with sepsis. The interaction of GM alteration with prolonged hospital stays, broad-spectrum antibiotics usage, and nutrition types has been shown in ICU patients with sepsis [4]. GM alteration can be expected in these conditions. However, therapeutic manipulations based on microbiota may not be desirable in this stage according to the condition of the ICU patients. There is limited knowledge about the state of GM, especially their metabolites SCFAs, in the early stages of sepsis or in non-ICU patients with sepsis. In this situation, the variables affecting GM are limited; therefore, it may be reasonable to judge the alteration in GM in sepsis patients for any manipulation of microbiome-based therapy in the future. This study aimed to evaluate some key GM members, bacterial pathogens, and main SCFAs in non-ICU patients with sepsis in comparison to the control group.

## 2 Methods

### 2.1 Study design and subjects

In this case-control study, 45 stool samples with positive blood cultures of non-ICU patients with sepsis were included as the case group, and 15 healthy cases were selected as the control group. Two groups were matched based on gender and age variables since these variables affect the gut microbiota. Therefore, one healthy control person was selected for three patients by matching age and gender. Stool samples were collected from Tabriz University of Medical Sciences educational hospitals from October 2021 to August 2022. The inclusion criteria of sepsis patients were chosen based on the criteria confirmed for non- ICU patients with sepsis [12]. For this purpose, non-ICU patients with specific causative infectious agents according to positive blood cultures and some laboratory criteria were selected as the potential for the onset of sepsis. QSOFA (quick Sequential Organ Failure Assessment) criteria are designed for non-ICU patients; therefore, patients were evaluated based on these criteria (respiratory rate ≥22 breaths/min, systolic blood pressure ≤100 mmHg, and altered mental status ≤13). Patients with ≥2 qSOFA points were included in the study. Some underlying factors and disorders can affect the intestinal microbiota even in non-sepsis patients or they can be a risk factor for the onset of sepsis; as a result; exclusion criteria for the patient group included critically ill patients with sepsis in the ICU, hospitalization for >11 days, immunocompromised, cancer, probiotic use, pregnancy, diabetes, and organ transplantation. In addition, the exclusion criteria for the control group were cases without any underlying disease, un hospitalization, the use of antibiotics, and probiotics up to 2 months before sampling. Informed consent was obtained from all participants in this study and approved by the Ethics Committee of Tabriz University of Medical Sciences (Ref. No.: IR. TBZMED.REC.1400.328).

### 2.2 Sample preparation and DNA isolation

Stool samples were collected from eligible participants and stored at −80 °C in an ultra-freezer. For molecular tests, DNA was extracted from faecal by Sambio™ Stool DNA Extraction Kit (Azma gene company, Iran) according to the manufacturer's recommendation. The purity of DNA was determined by UV methods (NanoDrop, Epoch, BioTek, USA). The DNA concentration was normalized for all samples to 30 ng/μL and stored at −20 °C until to do the Real-Time PCR assay.

### 2.3 Faecal microbiota analysis by real-time PCR

The bacterial relative numbers in the two groups were assessed by the Livak method [13] in real-time PCR, using the specific primers of each bacterium (Table 1). The Real-time PCR assays were conducted in 72-well of 100 μL strips with SYBR green Master Mix without ROX (Ampliqon, Denmark) on Rotor-Gene RG-6000 Real-Time PCR Analyzer (Corbett, Australia) as follows: 1 μL (10 pM) of forward and reverse primer, and template DNA, 10 μL of 2× Master Mix, and 8 μL deionized water with 20 μL final volume for each reaction. Amplification conditions were 95 °C for 15 min as the hold step to hot spot Taq polymerase reaction, 95 °C for 20 s for initial denaturation, 40 cycles for annealing at 60 °C for 20 s, and 72 °C for 30 s for the extension as well as 72 °C for 10 min set for melt curve. Samples without template DNA were used as a negative control.

**Table 1:** Characteristics of primers of studied bacteria for real-time PCR.

Organisms	Primer	Products Size	Target gene
Enterobacteriaceae	F: GCGGTAGCACAGAGAGCTT R: GGCAGTTTCCCAGACATTACTCA	69 bp	16 S rRNA
*Bifidobacterium* sp	F: CGGGTGAGTAATGCGTGACC R: TGATAGGACGCGACCCCA	139 bp	16 S rRNA
*Bacteroides* sp	F: TGGTAGTCCACACAGTAAACGATGA R: CGTACTCCCCAGGTGGAATACTT	94 bp	16 S rRNA
*S. aureus*	F: AGCCAAGCCTTGACGAACTAAAGC R: GCGATTGATGGTGATACGGTT	279 bp	*nuc*R
*F. prausnitzii*	F: CCCGGCATCGGGTAGAG R: GGACGCGAGGCCATCTC	55 bp	16 S rRNA
*Lactobacillus* sp.	F: GCTAGGTGTTGGAGGGTTTCC R: CCAGGCGGAATGCTTAATGC	63 bp	16 S rRNA
Universal	F: TGGAGCATGTGGTTTAATTCGA R: TGCGGGACTTAACCCAACA	159 bp	16 S rRNA

F: forward, R: reverse.

### 2.4 Faecal SCFAs extraction

The extraction of faecal SCFAs and their analysis by HPLC with some changes were performed as previously explained [14]. Briefly, 300 mg of stool sample was homogenized with 1 mL of deionized distilled water and centrifuged at 12,000 rpm for 10 min 100 μL of HCl (37%) was added to the supernatant, and it was extracted on the rotator by adding 5 mL of diethyl ether for 20 min. After centrifugation at 3500 rpm for 5 min, the organic phase was mixed with 500 μL of NaOH 1 N. The centrifugation was repeated for 5 min at 3500 rpm, and 100 μL of HCl (37%) was added to the aqueous phase and vortexed. In the last step, 10 μL of this solution was injected into HPLC after filtration.

### 2.5 High-performance liquid chromatography (HPLC)

SCFAs were analyzed by the Smart line Knauer HPLC system (Germany) including a pump (Smartline pump 1000, Knauer), a spectrophotometric UV detector adjusted at the wavelength of 210 nm for analyte detection (Smart line UV detector 2600, Knauer) and a 20 μL Knauer loop injector. The chromatographic system was controlled by the EZChrom Elite system component provided by Knauer, and data processing was conducted using the Chromgate software. The analytes were separated using a C18 column (250 mm × 4.6 mm ID, 5 μm) (Knauer, Germany). A solution of NaH_2_PO_4_ 95% (20 mM, with a pH adjusted to 2.5) and acetonitrile 5% were used as the mobile phase. Acetic, propionic, and butyric acid standards were utilized to identify and determine their concentration in the samples of patients with sepsis and healthy individuals. For this purpose, serial dilutions of standards were prepared according to the values of the areas under the curve of the clinical samples.

### 2.6 Data analysis

Descriptive statistics were performed to assess the prevalence of bacteria isolated from patients with positive blood cultures, demographic, and clinical variables. In the analytical statistics section, based on the normality or abnormality of the data, the *t*-test and Mann-Whitney *U* test were carried out to compare the mean relative amounts of bacteria and the concentration of short-chain fatty acids in the two groups. The mean ± standard deviation was used to report quantitative values. P value < 0.05% was considered a significant relationship. All statistical tests were performed using version 9.4.1 of GraphPad Prism.

## 3 Findings

### 3.1 Characteristics of descriptive statistics

The demographic, clinical, and laboratory information of the non-ICU patients with sepsis and healthy groups are summarized in Table 2. No significant difference was observed between age and sex variables in the two groups. Most of the positive blood culture samples were isolated from the infectious ward (40%).

**Table 2:** The demographic, clinical, and some laboratory information of the patients with sepsis and healthy groups.

Variables	Patients with sepsis	Healthy groups	P value
Age (Y)	55.84 ± 15.84	53.60 ± 12.49	0.62
Gender (male/female)	23 (51.11%)/22 (48.89)	8 (53.33%)/7 (46.67%)	0.88
Hospitalization days	6 (2–11)	–	–
CRP, mg/L	106.15 ± 51.14	–	–
WBC, 10^9^/L	13.7 ± 4.04	–	–
**Inpatient ward**			
Infectious	18 (40%)	–	–
Nephrology	12 (26.7%)	–	–
Neurology	6 (13.3%)	–	–
Respiratory	6 (13.3%)	–	–
GI	3 (6.7)	–	–

CRP: *C*-Reactive Protein. WBC: white blood cells. GI: Gastroenterology.

**Fig. 1:**
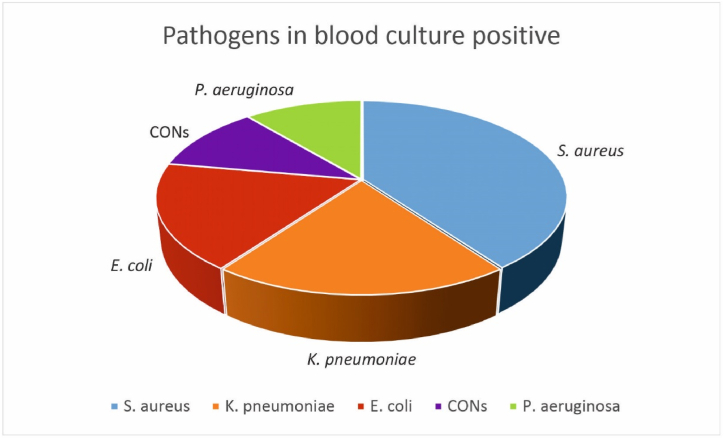
The abundance of bacteria isolated from blood cultures in patients.

The abundance of bacteria isolated from blood cultures in non-ICU patients with sepsis was *Staphylococcus aureus* (40%), *Klebsiella pneumonia* (20%), *Escherichia coli* (18%), *Pseudomonas aeruginosa* (11%), and Coagulase-negative Staphylococci (11%) (Fig. 1).

### 3.2 Alterations of GM

**Fig. 2:**
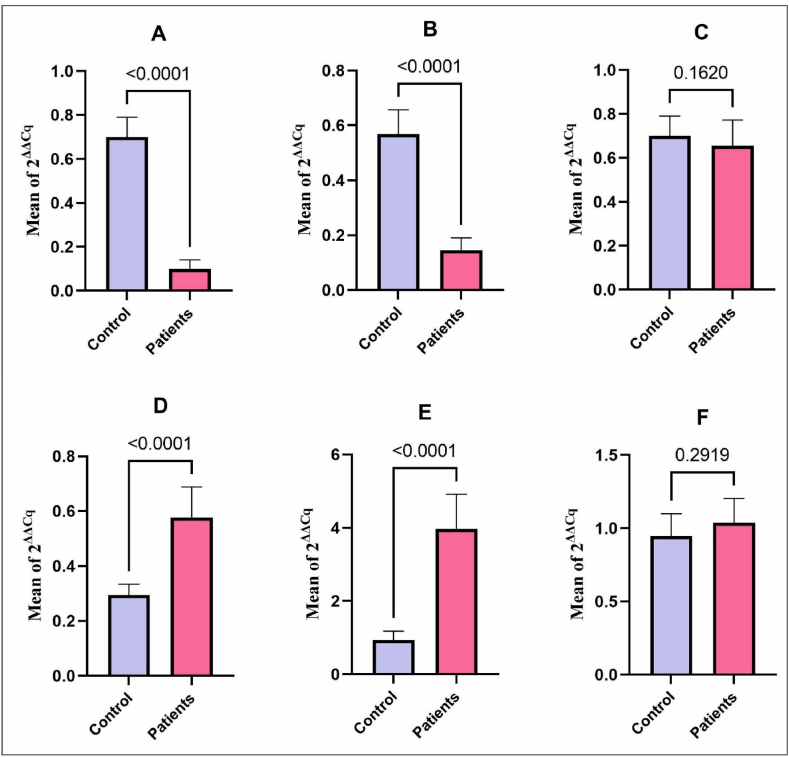
The bacteria relative amount in the patients with sepsis and the healthy group. A: *F. prausnitzii*, B: *Bifidobacterium* sp., C: *Lactobacillus* sp., D: Enterobacteriaceae, E: *Bacteroides* sp., F: *S. aureus*.

Real-time PCR analysis showed significant dysbiosis of some key GM members and pathogenic bacteria. The beneficial bacteria with a protective role, including *F. prausnitzii* (P = 0.0001) and *Bifidobacterium* sp. (P = 0.0001), significantly decreased in the patients with sepsis compared to the healthy group (Fig. 2 A and B). The levels of *Lactobacillus* sp. (P = 0.1620) decreased in the patient group, but no significant relevance was observed (Fig. 2C). In contrast, the pathogenic bacteria members of the Enterobacteriaceae (P = 0.0001) and *Bacteroides* sp. (P = 0.0001) increased significantly in the patients compared to the control groups (Fig. 2 D and E). Besides, the relative amount of *S. aureus* (P = 0.29) with a non-significant level was higher in patients compared to healthy groups (Fig. 2 F).

### 3.3 The characteristics of SCFAs

A proper level of short-chain fatty reflects a balance between the GM members; so, HPLC analysis showed a deeper dysbiosis of the intestinal microbiota than real-time PCR. By comparing the average level of short-chain fatty acids in the patients with sepsis and the healthy groups (Table 3.), it was found that the bacteria producing acetic, propionic, and butyric acid were different in the two groups. Acetic and propionic acid concentrations significantly increased in the control group than in the non-ICU patients with sepsis. The concentration of butyric acid decreased in the patients, but this change was not significant in the two groups (Table 3).

**Table 3:** The concentration of acetic acid, propionic acid, and butyric acid in the patients with sepsis and the healthy group.

SCFAs^a^	Patient with sepsis	Control group	p-value^b^
Acetic acid	31.21 ± 13.47	70.12 ± 24	0.0001
Propionic acid	1.81 ± 0.45	3.12 ± 0.88	0.0001
Butyric acid	1.27 ± 0.74	1.57 ± 0.33	0.0622

a : μmol/g stool sample.

b : Mann–Whitney *U* test.

## 4 Discussion

Some studies have shown altered gut microbiota in critically ill patients with sepsis [8,15]. Beneficial bacteria in the GM population may change early in sepsis and be a risk factor for the severity of this condition. SCFAs play an important role in host health. Studies on intestinal profile metabolites of GM such as SCFAs in non-ICU patients with sepsis are limited. Moreover, variables such as geographic location and race also affect the GM population. In the present study, some key GM members, bacterial pathogens, and SCFAs were evaluated in non-ICU patients with sepsis caused by bacteraemia.

In this investigation, significant alterations were observed in some GM members and the concentration of SCFAs. The relative amounts of *F. prausnitzii* and *Bifidobacterium* sp. Significantly decreased in the patients with sepsis compared to the healthy group. This finding is in agreement with previous studies in patients with sepsis [8,15]. An inflammatory situation has been shown in the intestines of patients with sepsis. Thus, it is possible to expect a decrease in the population of *F. prausnitzii*. The anti-inflammatory properties of *F. prausnitzii* have been confirmed by butyric and salicylic acid production [16]. On the other hand, the extracellular vesicles derived from this bacterium lead to the improvement of intestinal permeability by increasing the expression of ZO1, OCLN, and other genes [17]. *Bifidobacterium* species are among the most common bacterial genera in the role of probiotics [18]. Findings have shown Bifidobacterium species induce immunoregulatory responses and suppress inflammation [19]. An increase in intestinal permeability leads to the transfer of bacterial pathogens and lipopolysaccharide (LPS) as their structural components to the bloodstream in patients with sepsis. In this regard, a study showed that the administration of *Bifidobacterium* sp. Leads to a decrease in the level of LPS in the patient's blood plasma [20]. Furthermore, a negative relationship between *Bifidobacterium* sp. And inflammation markers such as CRP has been shown [8]. In contrast with previous findings, the beneficial role of *Lactobacillus* sp. In improving health was not confirmed in this study [21]. One of the most probable reasons for this discrepancy could be that our study has covered several species of *Lactobacillus* instead of one specific species.

The growth chance of opportunistic bacterial pathogens increases in GM with dysbiosis conditions. Under normal conditions, the dynamic GM prevents pathogenic bacteria growth by producing antimicrobial agents and occupying receptors [4]. According to this evidence, and in agreement with other findings, in this study, pathogenic bacteria members of the Enterobacteriaceae and anaerobic *Bacteroides* sp. Significantly increased in the sepsis patients [22,23]. The reason for this difference is probably the patient's condition related to GM, For instance, profound dysbiosis in critical ICU patients with sepsis versus relative dysbiosis in non-ICU patients with sepsis. Moreover, unlike Bacteroides sp. And Enterobacteriaceae, although Staphylococcus exists in the intestine, it is not a primary pathogen or colonizer bacteria in the intestinal, and its infection originates from other places such as skin and nasal from the nosocomial acquisition [24].

The results based on human evidence and probiotic interventions in mouse models strongly support the effect of SCFAs on improving the immune system and maintaining intestinal barrier integrity [25]. For instance, SCFAs can maintain intestinal barrier integrity through G protein-coupled receptors activation and histone deacetylases inhibition [26]. Moreover, the protective effect of SCFAs in several investigations has been revealed against infectious agents through direct or indirect mechanisms of action [27]. The main SCFAs in the intestine with 2–4 carbon atoms include acetic, propionic, and butyric acid. A series of complex metabolic pathways must be activated to induce the production of these short-chain fatty acids, and this function is performed through a healthy and dynamic GM. Therefore, in agreement with these findings, the low concentration of SCFAs can reflect dysbiosis and the absence of a dynamic microbiota. The evidence of our study showed a significant reduction in acetic and propionic acid concentrations (Table 3.). Acetic acid can enter the blood circulation and affect the T cells, PMN, and monocyte functions [28]. Moreover, the ability of acetic acid to produce different cytokines from immune cells and create a suitable ratio of oxidants and antioxidants has been shown [29]. As a result, acetic acid can contribute to the elimination of pathogens causing sepsis in patients. A specific mechanism of propionic acid performance has not yet been reported, but the relationship between propionic acid concentration with various diseases has been observed. The protective effects of propionic acid on blood pressure disorders, cardiac function, fibrosis, and cardiac hypertrophy were shown in an experimental study. It has also been found that propionic acid leads to increased T regulatory cell differentiation and efficiency. In agreement with the findings from the evaluation of propionic acid in critically ill patients with sepsis [30], our results showed a significant decrease in the concentration of this SCFA in patients with sepsis. Butyrate can affect the host receptor systems in the role of the signaling agent. For example, improving inflammation and regulating energy for enterocytes occur by binding butyrate and other short-chain fatty acids to G-protein-coupled receptors 41 and 43, the mechanism of which is through the production of glucagon-like peptide 1 (GLP-1) in the intestine [31]. Contrary to previous results, the butyric acid concentration in our study was not significant compared to the control group. The effects of butyrate deficiency in sepsis cannot be ignored because evidence has shown that butyrate leads to the induction of intestinal mucosa proliferation and the repair of the damaged epithelium [32]. There is a close relationship between *F. prausnitzii* and butyric acid production. As mentioned, *F. prausnitzii* significantly decreased among patients, but this trend was not observed with butyric acid. This discrepancy may be the result that *F. prausnitzii* is not the unique bacteria for producing butyrate. In this regard, other bacteria like *Roseburia*, *Megasphaera elsdenii*, *Caldocellum saccharolyticum*, *Eubacterium rectale*, and Clostridium spp. Can produce butyrate [33]. Therefore, other important producing butyrate bacteria may not have been destroyed at the onset of primary sepsis in this study. Because some variables like the hospitalization period, the type of patient's diet, and the use of broad-spectrum antibiotics can affect the GM. Overall, studies have shown that critically ill patients experience dysbiosis and, in parallel, a decrease in the concentration of SCFAs. Consequently, this situation can induce inflammation, cell apoptosis, diarrhea, and translocation of pathogenic bacteria [11,34].

In our study, it was found that there are significant changes in some key GM populations and short-chain fatty acids in patients with sepsis. The remarkable point is that this alteration occurred in the primary stage of non-ICU patients with sepsis, and these changes may be more profound in the critical phase of severe sepsis and septic shock situation. The alteration of GM and their metabolites in the present study may be a risk factor for inducing the incidence and severity of sepsis involvement compared to the healthy group. As in some studies, Gut microbiota alteration or dysbiosis has been considered a risk factor for sepsis. At this point in history, the impact of GM as a new organ on human health is undeniable. Further studies in this setting can help to identify the key altered GM members and their metabolites. Thus, the restoration of such changes may be very critical for patients. In addition, some studies have pointed out the adverse effects of increasing SCFAs and even some probiotic bacteria of GM; as a result, more studies are recommended for the safety of this strategy to improve health.

Our limitations in this research included the lack of access to sequencing-based methods for GM evaluation. Also, the inability to identify other metabolites of GM may be one of the other limitations.

## 5 Conclusions

Overall, our findings confirmed the occurrence of dysbiosis and some bacteria metabolite alterations in the intestinal of patients with sepsis compared healthy group. Due to the protective and anti-inflammatory role of *F. prausnitzii*, *Bifidobacterium* sp., and short-chain fatty acids, the modified microbiome and their metabolites may be helpful for the management of patients with sepsis. The efficacy of this strategy will be proven by conducting more studies to understand the GM profile in these patients.

## Author contribution statement

Edris Nabizadeh: Conceived and designed the experiments; Performed the experiments; Wrote the paper.

Javid Sadeghi: Conceived and designed the experiments; Analyzed and interpreted the data.

Mohammad Ahangarzadeh Rezaee: Analyzed and interpreted the data; Wrote the paper.

Hamed Hamishehkar: Contributed reagents, materials, analysis tools or data; Analyzed and interpreted the data.

Alka Hasani: Performed the experiments; Contributed reagents, materials, analysis tools or data.

Hossein Samadi Kafil: Analyzed and interpreted the data; Contributed reagents, materials, analysis tools or data.

Yaghoob Sharifi: Wrote the paper; Analyzed and interpreted the data.

Solmaz Asnaashari: Performed the experiments; Contributed reagents, materials, analysis tools or data.

Hiva Kadkhoda: Performed the experiments.

Reza Ghotaslou: Conceived and designed the experiments; Analyzed and interpreted the data; Wrote the paper.

## Data availability statement

Data will be made available on request.

## Additional information

No additional information is available for this paper.

## Funding

This study was supported by the Infectious and Tropical Diseases Research Center, 10.13039/501100004366Tabriz University of Medical Sciences Tabriz, Iran. [grant number 5/د/65618 and 6515/4/5].

## Declaration of competing interest

The authors declare that they have no known competing financial interests or personal relationships that could have appeared to influence the work reported in this paper.
